# Ketogal Safety Profile in Human Primary Colonic Epithelial Cells and in Mice

**DOI:** 10.3390/ph14111149

**Published:** 2021-11-11

**Authors:** Federica Sodano, Bice Avallone, Monica Tizzano, Chiara Fogliano, Barbara Rolando, Elena Gazzano, Chiara Riganti, Salvatore Magliocca, Mariarosaria Cuozzo, Stefania Albrizio, Antonio Calignano, Claudia Cristiano, Roberto Russo, Maria Grazia Rimoli

**Affiliations:** 1 Department of Drug Science and Technology, University of Torino, 10125 Torino, Italy; federica.sodano@unito.it (F.S.); barbara.rolando@unito.it (B.R.); 2 Department of Pharmacy, University of Naples Federico II, 80131 Naples, Italy; mariarosaria.cuozzo@unina.it (M.C.); salbrizi@unina.it (S.A.); antonio.calignano@unina.it (A.C.); roberto.russo@unina.it (R.R.); rimoli@unina.it (M.G.R.); 3 Department of Biology, University of Naples Federico II, 80126 Naples, Italy; bice.avallone@unina.it (B.A.); monica.tizzano@unina.it (M.T.); chiara.fogliano@unina.it (C.F.); 4 Department of Life Sciences and Systems Biology, University of Torino, 10123 Torino, Italy; elena.gazzano@unito.it; 5 Department of Oncology, University of Torino, 10126 Torino, Italy; chiara.riganti@unito.it; 6 Department of Pharmaceutical and Pharmacological Sciences, University of Padova, 35131 Padova, Italy; magliocca.s@gmail.com

**Keywords:** ketorolac, ketogal, histological evaluation, colonic cytotoxicity, mitochondrial oxidative stress

## Abstract

In our previous studies, a ketorolac–galactose conjugate (ketogal) showed prolonged anti-inflammatory and analgesic activity, causing less gastric ulcerogenic effect and renal toxicity than its parent drug ketorolac. In order to demonstrate the safer profile of ketogal compared to ketorolac, histopathological changes in the small intestine and liver using three staining techniques before and after repeated oral administration in mice with ketorolac or an equimolecular dose of its galactosylated prodrug ketogal were assessed. Cytotoxicity and oxidative stress parameters were evaluated and compared in ketorolac- and ketogal-treated Human Primary Colonic Epithelial cells at different concentrations and incubation times. Evidence of mitochondrial oxidative stress was found after ketorolac treatment; this was attributable to altered mitochondrial membrane depolarization and oxidative stress parameters. No mitochondrial damage was observed after ketogal treatment. In ketorolac-treated mice, severe subepithelial vacuolation and erosion with inflammatory infiltrates and edematous area in the intestinal tissues were noted, as well as alterations in sinusoidal spaces and hepatocytes with foamy cytoplasm. In contrast, treatment with ketogal provided a significant improvement in the morphology of both organs. The prodrug clearly demonstrated a safer profile than its parent drug both in vitro and ex vivo, confirming that ketogal is a strategic alternative to ketorolac.

## 1. Introduction

Non-steroidal anti-inflammatory drugs (NSAIDs) are the primary drugs of choice for the treatment of a variety of chronic and acute inflammatory conditions. In recent decades, the high incidence of chronic diseases, such as arthrosis, in the western world has led to an increase in the use of NSAIDs. Today, NSAIDs account for approximately 8% of total drug prescriptions [1].

However, NSAIDs are associated with several side effects [2], and much attention has been devoted to their toxic effects on the gastrointestinal (GI) tract, particularly the stomach, although a number of studies has demonstrated extensive damage to the intestine. In several cases, intestinal complications outnumbered gastric ones [3,4,5,6].

It is known that long-term use of NSAIDs can damage the small and large intestines in healthy subjects, causing bleeding and perforation, erosion, ulcers, and bowel obstruction [7,8]. This can lead to reduced intestinal absorption capacity and increased permeability [9,10]. In as many as 80% of users, chronic NSAID use can exacerbate pre-existing conditions, such as colitis and strictures, inflammatory bowel disease, irritable bowel syndrome, and diverticular disease. Proton-pump inhibitors (PPIs) are routinely co-prescribed with NSAIDs to prevent GI injury. However, while such co-administration leads to a reduction in gastric injury, it may worsen small bowel ulceration and bleeding [11,12]. This is because the intestinal damage induced by NSAIDs is more closely related to their enterohepatic circulation and the following repeated exposure of the intestinal epithelium to these drugs in the presence of bile [13]. In addition, chronic suppression of gastric acid by PPIs may trigger a bacterial overgrowth in the stomach and small intestine and consequently dysbiosis [13]. Dysbiosis, in turn, causes a significant increase in small intestinal permeability, which is considered the early event in the pathogenesis of NSAID enteropathy [13].

A variety of clinical studies has addressed NSAID-induced damage in the upper GI tract. This is particularly true for the small intestine because of its easy accessibility, which facilitates the diagnosis of damage. Upon endoscopic examination, acute bleeding and erosion of the gastroduodenal mucosa can be easily detected in most NSAIDs users [14]. However, NSAIDs can damage any area of the entire GI tract. No less than 40% of all NSAID-induced adverse events are in the lower GI tract, including the colon. These complications include increased permeability, mucosal inflammation, ulceration and hemorrhage, and strictures caused by fibrous diaphragms [15].

Hepatic injury is an additional side effect that has been poorly documented. The liver, because of its significant role in drug metabolism, often suffers from toxic side effects. In fact, hepatotoxicity is the most common reason for non-approval, restriction of indications, and sometimes withdrawal of drugs [16]. NSAIDs present a different spectrum of liver damage, ranging from asymptomatic and transient hypertransaminasemia to fulminant hepatic failure. Interestingly, NSAID-induced hepatotoxicity may be associated with six- to nine-fold greater liver damage in patients concomitantly taking other potentially hepatotoxic drugs (e.g., amoxicillin–clavulanic acid, proton pump inhibitors, phenobarbital, isoniazid) [17].

The main mechanism underlying the occurrence of the aforementioned NSAID lesions is the inhibition of cyclooxygenases (COX-1 and COX-2), which are the enzymes involved in the conversion of arachidonic acid into prostaglandins and other lipid mediators. They differ in their regulatory mechanisms and tissue distribution. In detail, COX-1 produces prostaglandins that regulate normal cellular processes, whereas COX-2 can be induced by inflammation or growth factors. Prostaglandins are responsible for restoring intestinal homeostasis. Indeed, they facilitate the regeneration of the intestinal epithelium [18]. NSAID-induced inhibition of prostaglandin secretion may lead to the collapse of the mucosal defense system and thus leave the intestine more exposed to a variety of insults. In general, NSAIDs selectively inhibit COX-2 as opposed to COX-1 for pain-relieving effects (anti-hyperalgesia).

The COX-independent topical effect of NSAIDs on the small bowel epithelium is another mechanism underlying NSAID injury. This activity plays an important role during the early stage of injury and is caused by mitochondrial impairment [19]. Somasundaram et al. have reported morphological changes of mitochondria in the initial process of small bowel ulceration after oral treatment with indomethacin [20]. Specifically, mitochondrial vacuolization 1 h after indomethacin treatment, mitochondrial swelling, and loss of cristae at 2 h were detected by electron microscopy [20,21]. These changes in mitochondria morphology could be attributable to the activity of NSAIDs to inhibit electron transport and uncouple the oxidative phosphorylation process. Indeed, in vivo treatments with certain NSAIDs caused the opening of the channel called the mitochondrial permeability transition pore (PTP) and thus an increase in permeability across the mitochondrial membrane [22,23,24]. In the case of diclofenac and ketorolac, the mitochondrial permeability transition occurred only under oxidative stress and in a calcium-dependent manner [25]. Despite the large number of studies, the exact biochemical mechanism by which NSAIDs inhibit, or uncouple, mitochondrial oxidative phosphorylation remains unclear.

Treatment with NSAIDs may also trigger a decrease in oxygen uptake [26]. This additional alteration, along with the NSAID-induced increase in mitochondrial permeability and changes in mitochondria morphology, appears to be mediated by the production of reactive oxygen species (ROS) [27]. ROS are likely produced by activation of xanthine oxidase and infiltration of neutrophils into the mucosa [27]. An in vitro study showed that an increase in xanthine oxidase activity with a concomitant decrease in xanthine dehydrogenase activity is associated with hyperproduction of superoxide anion, resulting in oxidative stress [28,29].

In this scenario, the exploitation of ketogal (ketorolac-d-galactos-6′-yl ester), the conjugate of a well-known drug, ketorolac, with a non-toxic molecule, galactose, may be an effective solution in terms of safety [30]. In order to investigate whether similar alterations to those described occurring in the small intestine also occur in the lower GI tract, we evaluated the cytotoxicity of ketorolac and ketogal against Human Primary Colonic Epithelial (CoEpi) cells. Another aim of the present study was to perform histopathological and morphological evaluations on the small intestine and liver, under the same experimental conditions used for the stomach and kidneys and described in our recent work [31].

## 2. Results

### 2.1. Stability of Ketogal and Ketorolac in PBS and CoEpi Cell Medium

**Figure 1:**
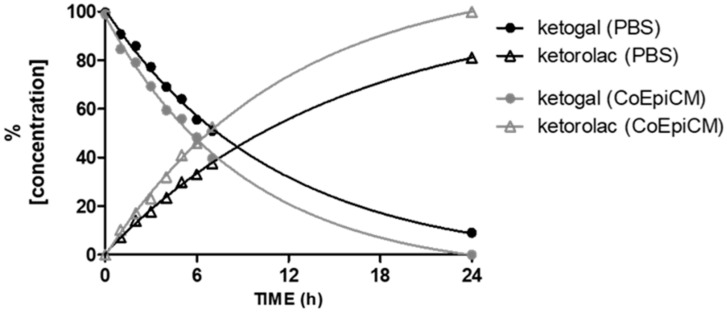
Stability of ketogal in PBS and CoEpi cell medium. Results are expressed as mean values (*n* = 3).

Prior to the in vitro experiments, the chemical stability of ketogal and ketorolac was evaluated at 37 °C, both at pH 7.4 in PBS and in the cellular medium used for human CoEpi cells, as reported in Figure 1. These data allowed us to determine the extent of release of the parent drug, in our case ketorolac, from the galactosylated prodrug, ketogal, as a result of the susceptibility of the ketorolac–galactose conjugate to hydrolysis. Ketorolac was still stable after 24 h of incubation in both experimental conditions (data not shown). In addition, ketogal demonstrated good stability both under simulated physiological conditions and in cellular medium. Indeed, after 2 h of incubation, the percentage of ketogal remaining was about 78% in CoEpi cell medium and about 85% in PBS; ketorolac was the only hydrolysis product in all conditions (Figure 1) In detail, the half-life (t_½_) of ketogal in CoEpi cell medium was found to be 6.4 h.

### 2.2. Human CoEpi Cell Viability

**Figure 2:**
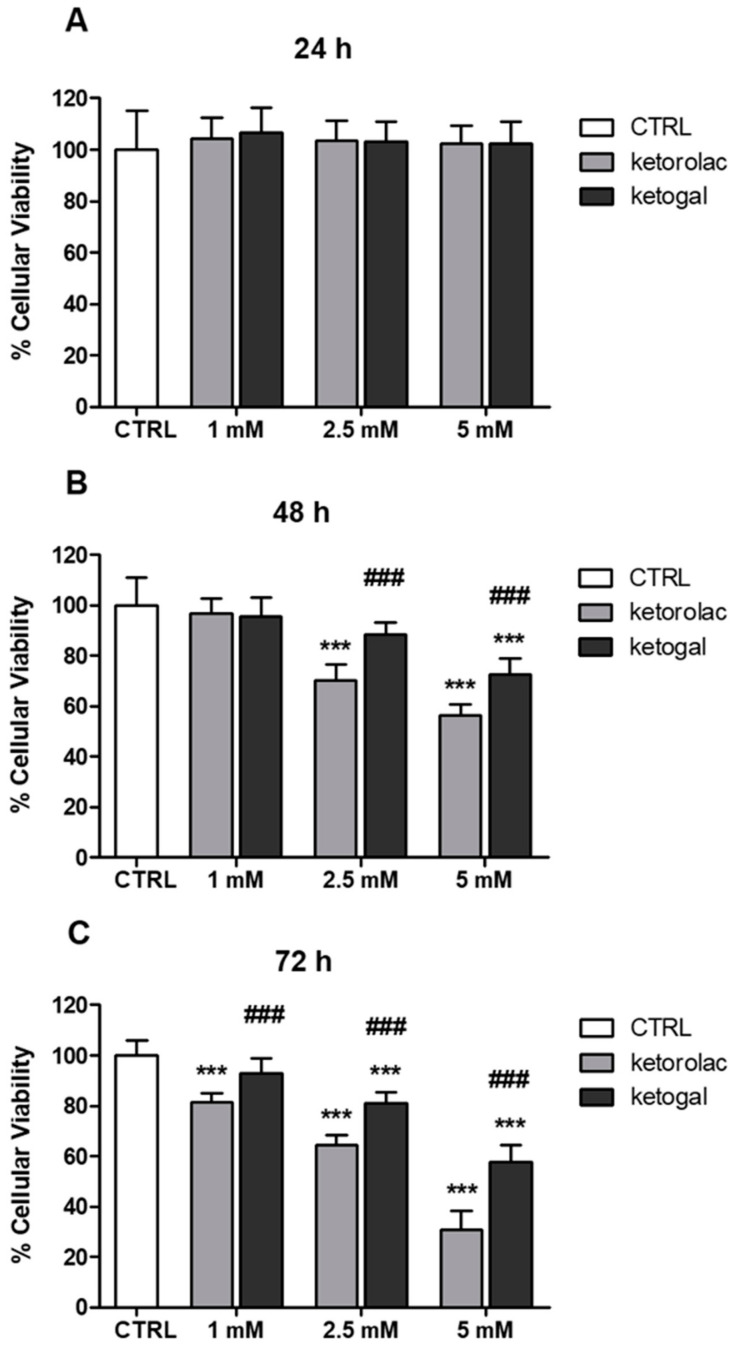
Effect of ketorolac and ketogal on the mitochondrial activity of human CoEpi cells. Cells were incubated for 24 (**A**), 48 (**B**), and 72 (**C**) h either in the absence (CTRL) or presence of 1, 2.5, or 5 mM ketorolac or ketogal. Data are presented as means ± SEM (*n* = 3). Significance of treated (ketorolac or ketogal) versus respective untreated cells: *** *p* < 0.001. Significance of ketogal vs. ketorolac: ### *p* < 0.001.

In order to predict the toxicity of ketogal in the colon, compared to ketorolac, human CoEpi cells were incubated at different times and at different concentrations of the parent drug and prodrug. The choice of three time points, 24, 48, and 72 h, was due to the remarkable and sustained anti-inflammatory activity of ketogal, as demonstrated in our past works [31,32]. The three concentrations were selected using a previous dose–response curve of CoEpi cells incubated with ketorolac and ketogal, where we observed no reduced viability in the range of 5–500 µM for either drug (data not shown). We therefore decided to start evaluating the lowest concentration of ketorolac that induced cytotoxicity, i.e., 1 mM after 72 h, and two higher concentrations (2.5 and 5 mM). The WST-1 assay was used to assess cell viability as it is related to mitochondrial activity and thus cellular metabolism (Figure 2A–C). As shown in Figure 2A, neither ketorolac nor ketogal displayed any significant toxicity after 24 h of incubation. In contrast, after 48 h of incubation (Figure 2B), both compounds exhibited a cytotoxic effect; starting at a concentration of 2.5 mM, the parent drug caused the death of about 30% of the cells, while the prodrug induced only a 10% reduction in cell viability under the same conditions. The cytotoxicity trend was dose-dependent. After 72 h of incubation (Figure 2C), significant ketorolac-induced cytotoxicity was observed at the lowest dose (1 mM), whereas ketogal did not reduce mitochondrial activity compared to untreated cells at this concentration. At concentrations of 2.5 and 5 mM, the number of viable cells was always significantly lower with ketorolac than with ketogal.

### 2.3. Quantitative Evaluation of Ketogal and Ketorolac in CoEpi Cells

**Figure 3:**
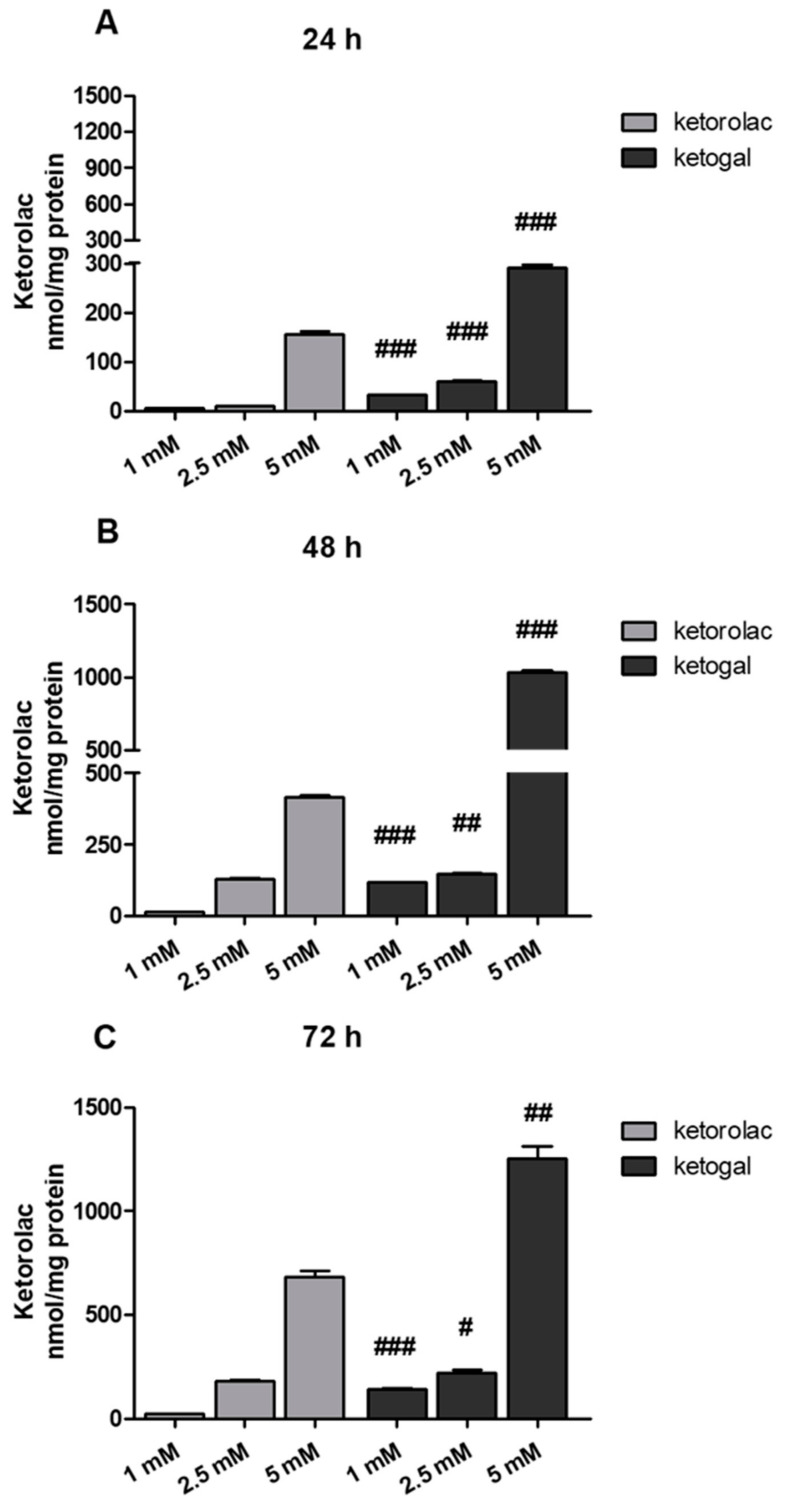
Amount of ketorolac in cell extracts of CoEpi cells incubated for 24 (**A**), 48 (**B**), or 72 (**C**) h in the presence of either 1, 2.5, or 5 mM ketorolac or ketogal. Data are presented as means ± SEM (*n* = 3). Significance of ketogal vs. ketorolac: # *p* < 0.05; ## *p* < 0.01; ### *p* < 0.001.

In order to confirm that the presence of a carrier, D-galactose, improves not only the stability and toxicological profile, but also the drug uptake of ketorolac, the amounts of ketorolac and ketogal in cell lysates were evaluated (Figure 3A–C). The results are expressed in terms of ketorolac concentration. In the case of ketogal, the concentration of ketorolac refers to that released after enzymatic hydrolysis. As can be seen, at each time point (24, 48, and 72 h) and at each concentration (1, 2.5, 5 mM), the amount of ketorolac released from ketogal was always higher than that detected in cells incubated with ketorolac (Figure 3A–C). This is further confirmation that the prodrug approach, using galactose as the carrier, provides several advantages, including an increase in the effective concentration of the drug in the target cells.

### 2.4. Mitochondrial Depolarization and Oxidative Parameters

**Figure 4:**
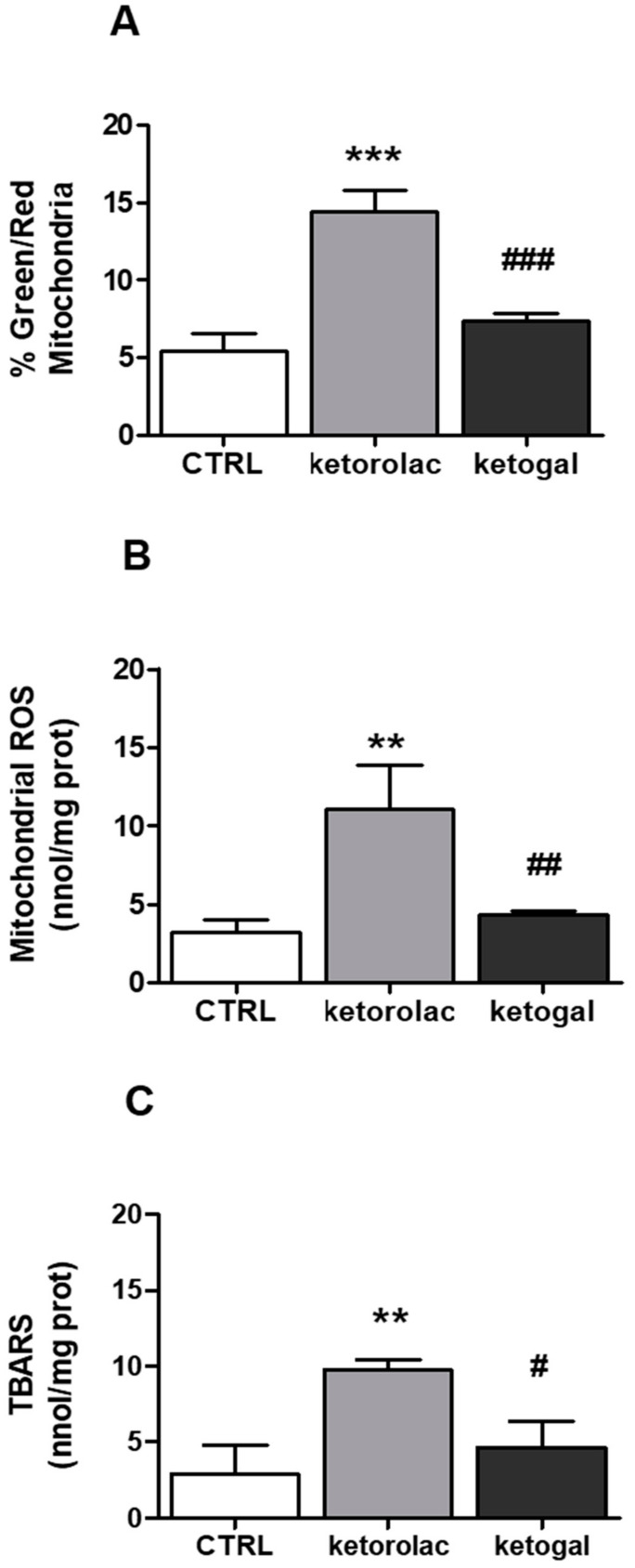
(**A**) Mitochondrial membrane potential was measured fluorometrically in triplicate using the JC-1 probe. Data are presented as means ± SEM *(n* = 3) of the percentage of green-fluorescent/red-fluorescent mitochondria. Significance of treated (ketorolac or ketogal) versus respective untreated cells: *** *p* < 0.001. Significance of ketogal vs. ketorolac: ### *p* < 0.001. (**B**) Mitochondrial ROS level was measured fluorometrically in triplicate using the MitoSOX Red probe. Data are presented as means ± SEM (*n* = 3). Significance of treated (ketorolac or ketogal) versus respective untreated cells: ** *p* < 0.01. Significance of ketogal vs. ketorolac: ## *p* < 0.01. (**C**) Mitochondrial TBARS levels were measured spectrophotometrically in triplicate using the TBARS assay kit. Data are presented as means ± SEM (*n* = 3). Significance of treated (ketorolac or ketogal) versus respective untreated cells: ** *p* < 0.01. Significance of ketogal vs. ketorolac: # *p* < 0.05.

In order to understand whether the cytotoxic effects induced by ketorolac in human CoEpi cells were caused by the mitochondrial alterations that NSAIDs generally induce in the GI tract, mitochondrial depolarization was assessed by JC-1 staining. The results were expressed as a ratio of green (i.e., damaged)/red (i.e., not damaged) mitochondria, which is considered an index of mitochondrial depolarization and permeability transition. The incubation time chosen was 48 h at 2.5 mM concentration. In fact, as shown in Figure 2B, the first sign of cytotoxicity with both drugs occurred under these conditions. In line with previous results reporting mitochondrial damage elicited by NSAIDs [22], ketorolac induced significant mitochondrial depolarization. As shown in Figure 4A, mitochondrial depolarization in ketorolac-treated CoEpi cells was three times higher than that in untreated cells. On the other hand, ketogal revealed no significant differences compared to control samples. Therefore, in contrast to its parent drug, ketogal did not provoke any change in depolarization and permeability across the mitochondrial membrane. Mitochondrial oxidative stress parameters were then analyzed, including ROS and TBARS. As shown in Figure 4B, the amount of ROS detected in samples incubated with ketorolac was remarkably high. On the other hand, ketogal did not display any difference compared to the control. Thus, ketogal did not alter the level of ROS that the mitochondria of human CoEpi cells physiologically generate. The concentration of TBARS was higher in the mitochondria samples treated with ketorolac than in those treated with ketogal (Figure 4C). Indeed, the value of TBARS detected in ketogal samples was comparable to that of untreated ones.

### 2.5. Intracellular Glutathione Measurement

**Figure 5:**
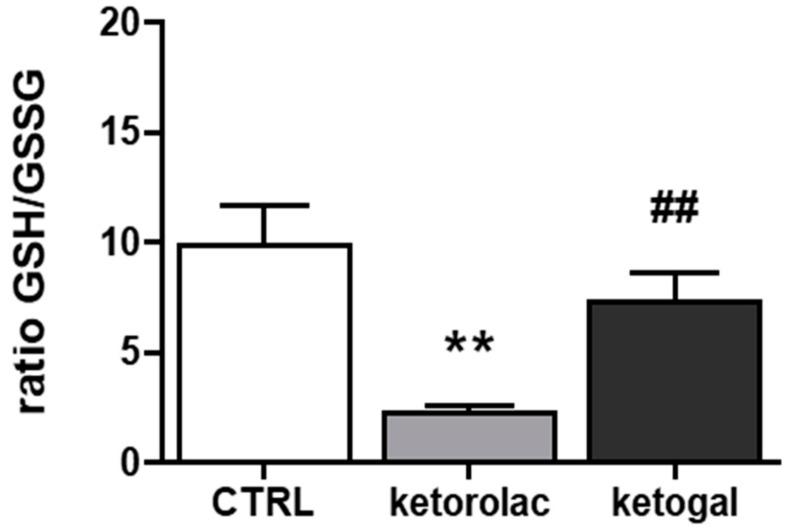
Glutathione measurement. GSH and GSSG were measured spectrophotometrically in triplicate in the cell lysates and expressed as nmol of glutathione/min/mg cellular protein. Data are presented as means ± SEM (*n* = 3) of GSH/GSSG ratio. Significance of treated (ketorolac or ketogal) versus respective untreated cells: ** *p* < 0.01. Significance of ketogal vs. ketorolac: ## *p* < 0.01.

The higher amount of ROS and TBARS at the mitochondrial level observed in the ketorolac-treated samples was in parallel with a lower GSH/GSSG ratio in the whole cells. As shown in Figure 5, the GSH/GSSG ratio was higher for ketogal compared to ketorolac and, at the same time, comparable to that of the control samples. This confirms that ketogal undoubtedly has a safer profile than its parent drug.

### 2.6. Evaluation of Small Intestine and Liver Toxicity after Repeated Oral Administration of Ketorolac and Ketogal

**Figure 6:**
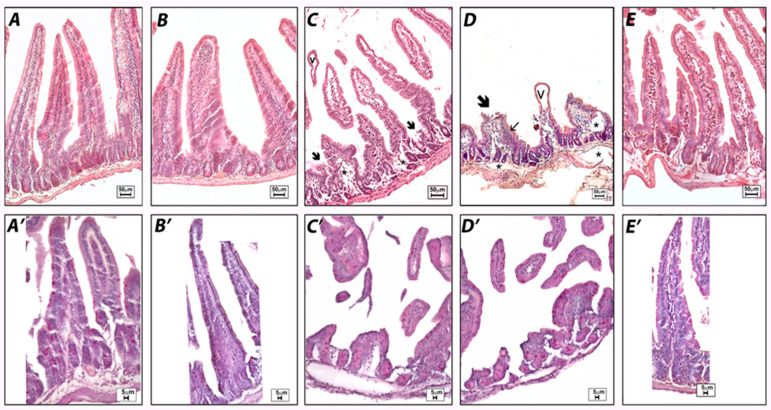
Histological observations of the different treatments on mouse small intestines. Hematoxylin–eosin (**A–E**) and PAS staining (**A′**–**E′**). Untreated (**A**) and vehicle (**B**) samples show normal small intestine morphology. Indomethacin (**C**) and ketorolac (**D**) samples demonstrate severe subepithelial vacuolations (v), eroded villi (arrow) with inflammatory infiltrates (small arrow), and edematous areas (*) with also a reduced villi length. The ketogal (**E**) sample demonstrates an improvement in intestine morphology. No significant changes in the production of mucus can be detected in any of the samples observed by PAS (6**A′**–**E′**).

**Figure 7:**
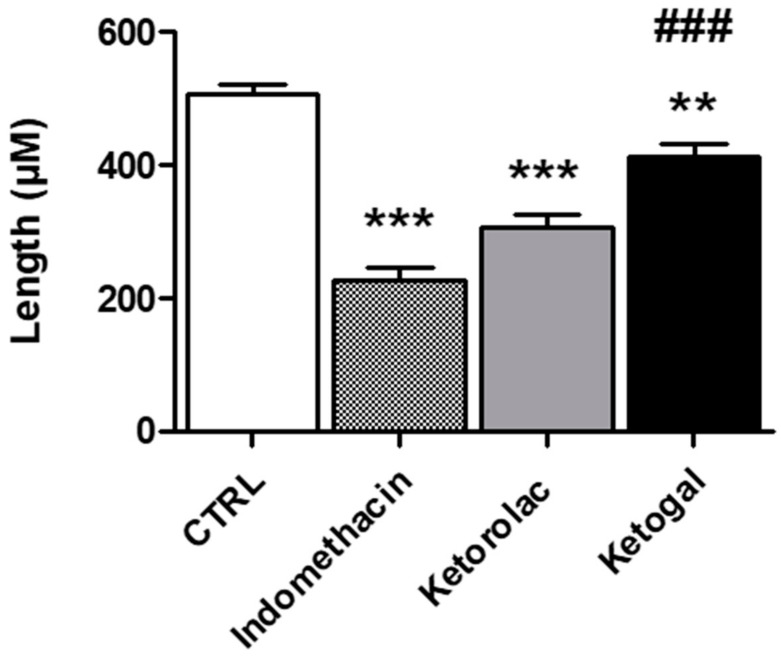
Effect of indomethacin, ketorolac, and ketogal on intestinal villi length (µm). Data are presented as means ± SEM (*n* = 3). Significance of indomethacin, ketorolac, or ketogal versus untreated: ** *p* < 0.01; *** *p* < 0.001. Significance of ketogal vs. ketorolac: ### *p* < 0.001.

In the intestine, hematoxylin–eosin-stained sections showed that the duodenum was arranged normally in the untreated and vehicle samples (grade 0): the mucosa, consisting of a single layer of epithelial cells, the lamina propria, and the muscularis mucosae; the sub-mucosa; the muscularis propria; and the serosa (Figure 6A,B). Enterocytes with uptake function, and goblet cells, which produce mucus, were well organized along the villi (Figure 6A′,B′). Mice treated with indomethacin, used as a positive control, and ketorolac showed compromised villi with subepithelial space and subepithelial vacuolation, erosion with loss of enterocytes, and goblet cells (grade 3) with presence of inflammatory infiltrates (Figure 6C,D). Deterioration of the villi was observed not only with alterations in their morphology but also with the halving of their lengths (Figure 7). In addition, edematous areas located in the mucosa and submucosa were observed (Figure 6D). Sections of the small intestine of ketogal-treated mice showed restoration of normal histology and the length of the villi, which appeared to be similar to that of untreated animals (Figure 6E). After treatment, neither inflammatory infiltrate nor erosion was evident (grade 2). On the other hand, PAS staining did not reveal substantial changes in mucus production in all observed specimens (Figure 6A′–E′).

**Figure 8:**
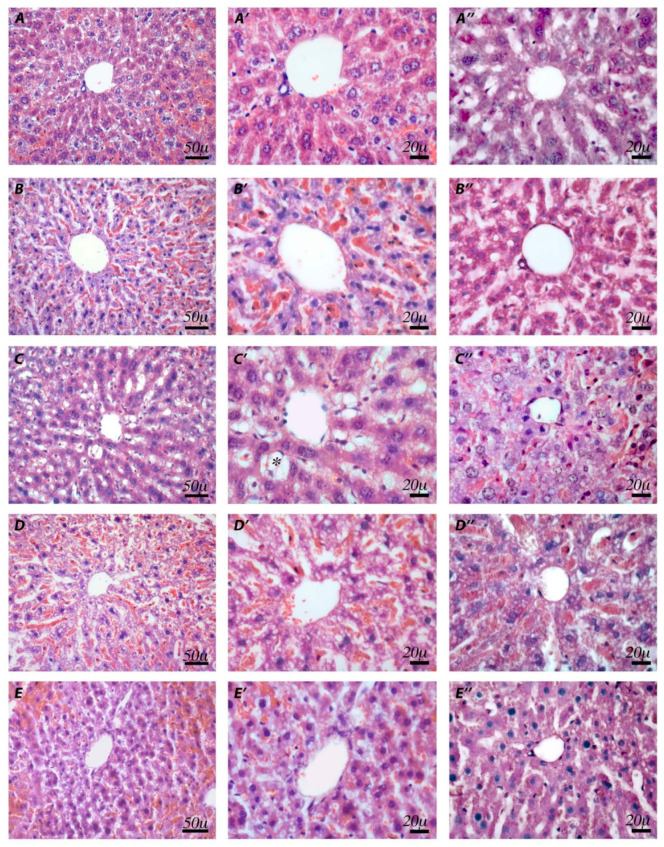
Histological observations of different treatments in mouse livers. Hematoxylin–eosin (**A**–**E′**) and PAS staining (**A″**–**E″**). Untreated (**A**,**A′**) and vehicle (**B**,**B′**) samples show normal organization of hepatocytes and sinusoids. Indomethacin (**C**,**C′**) samples demonstrate dilatations of the sinusoids and hydropic hepatocytes with displacement of nuclei (*) of several hepatocytes. In ketorolac (**D**,**D′**) samples, a foamy cytoplasm appearance is evident in the hepatocytes. Ketogal (**E**,**E′**) samples show a general improvement in hepatic tissue histology. PAS staining shows no substantial changes in the vehicle (**A″**), compared to the untreated (**B″**) samples. In indomethacin (**C″**) and ketorolac (**D″**) samples, a decrease in positivity is detectable. Ketogal (**E″**) samples show a slight improvement in terms of positivity.

Regarding the liver, with histological investigations of the untreated samples after hematoxylin–eosin staining, both the lobule and the portal triad showed typical tissue morphology with regular-sized hepatocytes and normal sinusoids (Figure 8A–A′). Histological evaluations of the vehicle samples displayed a regular shape, although the sinusoids were slightly dilated, which was likely due to fasting (Figure 8B–B′). Indomethacin-treated mice showed partial degeneration of liver structures, such as dilation of the sinusoids and macrovescicular hepatocytes, which appeared swollen or hydropic with peripheral displacement of the nuclei, especially around the central vein (Figure 8C–C′). However, treatment with ketorolac resulted in impaired sinusoidal spaces and hepatocytes with microvescicular steatosis characterized by foamy cytoplasm (Figure 8D–D′). Despite vacuolation of hepatocytes in some areas, a general improvement in tissue histology was noted in samples from ketogal-treated mice, with recovery of normal hepatocyte morphology (Figure 8E–E′).

PAS staining, which highlights mucopolysaccharides, showed a slight reduction in positivity in the vehicle compared to the untreated samples (Figure 8A″–B″), a significant decrease in positivity after indomethacin and ketorolac treatment (Figure 8C″–D″), and a slight improvement after ketogal treatment (Figure 8E″). Moreover, PAS staining was negative for the content of the numerous vesicles found in both indomethacin and ketorolac treatments.

**Figure 9:**
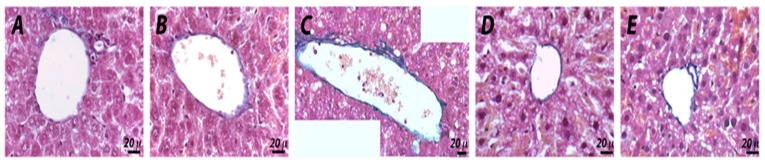
Histological observations of different treatments in mouse livers. Masson staining (**A**–**E**). In untreated and vehicle (**A**,**B**) samples, the regular presence of blue stained collagen fibers is observed. Indomethacin (**C**) samples show an increase in the collagen fibers below the endothelium of the portal and the central vein. In ketorolac (**D**) and ketogal (**E**) samples, no sign of fibrosis can be detected.

Masson staining showed that there was a regular presence of collagen beneath the endothelium of the portal vein and central vein in both untreated and vehicle-treated samples (Figure 9A,B). An increase in collagen fibers was observed in the sections of the indomethacin-treated mice (Figure 9C). On the other hand, no sign of fibrosis was detected in samples treated with ketorolac or ketogal (Figure 9D,E).

## 3. Discussion

Chronic treatment with ketorolac causes multiple side events despite its outstanding pharmacological profile. We have previously shown that esterification of ketorolac with a simple and natural carrier, such as D-galactose, improves its safety profile [31,32]. In particular, repeated administration of ketogal exerted significant pharmacological activity that was similar to that of its parent drug, ketorolac, but was less ulcerogenic and caused less renal toxicity [31].

In order to predict the effects on the lower GI tract, including the colon, we demonstrated the lower cytotoxicity of ketogal against human CoEpi cells. By increasing incubation times (in particular after 48 and 72 h) and drug dosing, the difference in toxicity between the parent drug and the prodrug became increasingly apparent. Moreover, at the same dosage, the amount of ketorolac actually delivered into human CoEpi cells was less than the amount of ketorolac resulting from hydrolysis of the prodrug. Therefore, ketogal also displayed improved uptake compared to ketorolac.

Mitochondrial oxidative stress parameters were measured, and changes in mitochondrial depolarization and permeability were explored with the aim of investigating whether ketorolac causes its toxic effects in human CoEpi cells through mitochondrial dysfunction, as previously reported [25,26,27], and comparing it with ketogal. Human CoEpi cells were chosen as an in vitro model for this study. This cell type is an excellent system for studying the impact of drugs on the human colon, such as NSAIDs. It has similar characteristics to the human primary cell monolayer system used by Bhatt et al. [33], and an equivalent ability to evaluate diclofenac induced-mitochondrial disorders in the small intestine. This model was not chosen by chance. In preliminary experiments, the toxicity of ketorolac and ketogal was previously modelled in vitro using transformed cell lines, including Caco-2 cells (data not shown). In accordance with Larregieu et al. [34], we found a miscorrelation between widely used Caco-2 colon carcinoma cell lines and CoEpi cells. This is due to the fact that the normal intestine, both the upper and lower tracts, differs from cancer cell lines, especially with regard to its metabolic activity. Cancer cells are known for their high metabolic rate and mitochondrial activity, which places limitations on the use of Caco-2 cells in studying the effects of NSAIDs on mitochondria [34]. For this reason, ketorolac- and ketogal-induced mitochondrial disorders were evaluated in a more physiological model of non-transformed colonic epithelial cells.

A significant increase in mitochondrial ROS levels was observed after incubation with ketorolac. This increase was paralleled to typical signs of oxidative damage, such as increased lipid peroxidation and a decrease in the intracellular GSH/GSSG ratio. The data were completely different for ketogal. At the same dose and incubation time, ketogal displayed no significant differences, compared to the control sample, in ROS and TBARS evaluations, or in the GSH/GSSG ratio measurements. Consequently, the lower viability of CoEpi cells, after 48 h of incubation with ketorolac and at 2.5 mM concentration (Figure 2B), was likely caused by the increased production of ROS of mitochondrial origin. According to the literature [35], reactive species, such as O_2_^−^, H_2_O_2_, and OH^.^, generate perturbations at the mitochondrial level and also induce altered permeability. This was confirmed by a JC-1 assay. The depolarization of the mitochondrial membrane after incubation with ketorolac was three times higher than with ketogal and control samples. A change in mitochondrial depolarization leads to a change in pH and in the value of the potential gradient between the mitochondrial intramembranous space and the matrix [36]. After changing the value of the potential gradient, ATP cannot be produced due to the uncoupling between the electron transport chain and oxidative phosphorylation, leading to cell death. In the case of ketogal, the cells showed no altered mitochondrial polarization. We can speculate that the 10% reduction in cell viability observed after 48 h of incubation with 2.5 mM ketogal, compared to the 30% reduction caused by ketorolac, may be related to the different impact that the two drugs, ketorolac and ketogal, may have on mitochondrial homeostasis. Another possible mechanism by which ketogal caused 10% cell death in CoEpi cells may be due to ketorolac-induced damage resulting from hydrolysis of its galactosylated prodrug.

Once it was established that ketogal did not induce oxidative stress in the lower GI tract, consistent with most clinical studies of NSAIDs, we demonstrated the safer profile of ketogal compared with ketorolac in the other organs as well, particularly in the upper GI tract [3,7] and in the liver [16,17]. Consistently, we examined histopathological changes after repeated oral treatment in mice with ketorolac and ketogal, under the conditions previously adopted for the stomach and kidneys in our past work [31].

At the level of the small intestine, from a morphologic point of view, our data showed a significant alteration of the intestinal villi, as a result of the presence of edematous areas and erosion in samples treated with indomethacin and ketorolac. Damage to this type was not only related to a change in mucus production but also to the impairment of regular permeability. Indeed, it has been shown, especially in the case of indomethacin, that a change in the lipid content of the brush border membrane (BBM) can lead to an increase in permeability [27]. This increase in permeability due to NSAID exposure allows the entry of several aggressive luminal factors, such as bile, enzymes, and bacteria, and is considered an important event in the pathogenesis of NSAID-induced enteropathy and to cause ulcerogenic and erosive situations [6]. A considerable amount of data indicates that the modification of BBM, due to NSAID treatment, was mediated by oxidative stress [27]. Indeed, NSAIDs can damage the respiratory chain, resulting in mitochondrial dysfunction and free-radical formation, but can also contribute to neutrophil infiltration [19]. In agreement with these data, our results show significant alterations in both intestinal permeability and cell infiltration in mice treated with indomethacin and ketorolac. Intestinal villi were morphologically damaged, and their length was reduced. In contrast, edema, erosion and neutrophil infiltration were considerably lower after ketogal treatment, as was the length of the villi, which was comparable to that of untreated mice. With regard to mucus production, no significant differences were found in the tested samples. It is known that treatments with NSAIDs can cause disturbances in glycosylation, resulting in a loss of the glycocalyx layer. This could easily be explained by the widespread presence of erosion, caused by treatment with indomethacin and ketorolac, which impaired total mucus production. Completely different results were obtained with ketogal. In fact, an almost completely normal morphology was observed in mice treated with this prodrug.

Ketogal also appears to be safer for the liver than its parent drug. In particular, hepatocytes from mice treated with indomethacin and ketorolac displayed partial degeneration of the liver structure, including dilatation of the sinusoids and edema of several hepatocytes. Furthermore, NSAIDs are able to cause microvesiculation, which are often described as the beginning of hepatic steatosis [37,38]. Microvesicular steatosis can lead to excessive hepatic-fat deposition, increased ROS formation, mitochondrial dysfunction, and endoplasmic reticulum stress, inducing inflammation, cell death, and finally fibrosis [39]. In our experiments, repeated treatment with ketorolac and indomethacin induced evident microvesiculation. On the other hand, the absence of fibrosis and microvesiculation after chronic administration with ketogal demonstrated that only minor liver toxicity was present, despite its ability to accumulate in the liver, as reported in our previous work [32].

The results obtained for the small intestine, liver, and, previously, for the stomach and kidneys [31], demonstrate that ketogal undoubtedly has the potential to overcome most of the side effects associated with repeated administration of ketorolac. Indeed, its toxicological profile has consistently exceeded expectations in in vivo and ex vivo models [31,32]. The preservation of the pharmacological activity of the parent drug and its extended anti-inflammatory and analgesic properties are two additional strengths to indicate its safety.

## 4. Materials and Methods

### 4.1. Chemicals and Materials

Ketorolac and indomethacin were purchased from Sigma-Aldrich, Milan, Italy. Ketogal was synthesized as reported in Magliocca’s work [40]. Plasticware for cultures was from Falcon (Becton Dickinson, Franklin Lakes, NJ, USA). Proteins in cell extracts were evaluated with the BCA kit from Sigma Chemical Co. (St. Louis, MO, USA). If not specified differently, the reagents were obtained by Sigma Chemical Co.

### 4.2. Stability of Ketogal and Ketorolac in Phosphate Buffered Saline (PBS) and Colonic Epithelial (CoEpi) Cell Medium

Ketorolac or ketogal (both at 10 mM) in DMSO were added to PBS (pH 7.4, 50 mM) or to CoEpi cell medium. The resulting solutions (100 µM) were maintained at 37 ± 0.5 °C. At given time intervals, PBS solutions were directly analyzed by RP-HPLC (described below); in the case of cell medium solutions, at fixed time intervals, 300 µL of reaction mixture were withdrawn and added to 300 µL of CH_3_CN with 0.1% TFA. Samples were sonicated, vortexed, and then centrifuged for 10 min at 2150 g. The clear supernatants were filtered using 0.45 µm PTFE filters (Alltech) and analyzed via RP-HPLC (described below). The experiments were performed in triplicate. Pseudo-first-order half-times (t_½_) for the chemical hydrolysis of ketogal to ketorolac were calculated from the linear slope of the plot of the logarithm of the remaining compound against time.

### 4.3. Cells

Human Primary Colonic Epithelial (CoEpi) cells (CliniSciences, Guidonia Montecello, Italy) were cultured on polylysine-coated plates in Colonic Epithelial Cell medium (CellBiologics, Chicago, IL, USA), supplemented with the Human CoEpi cell medium Supplement Kit (CellBiologics, Chicago, IL, USA) as per the manufacturer’s instructions. Cells were maintained at 5% CO_2_ and 37 °C and were used between passages 2 and 5.

#### 4.3.1. Viability Assay

A total of 1 × 10^4^ cells, seeded in a 96-well white plate, was incubated with the compounds for 24, 48, or 72 h. Cell viability was assessed with the Cell Proliferation Reagent WST-1 (Sigma Chemical Co., St. Louis, MO, USA). The tetrazolium salt WST-1 is cleaved to formazan by mitochondrial dehydrogenases: an increase in the number of viable cells results in an increase in enzyme activity, which leads to an increase in the amount of formazan. The formazan produced is directly proportional to the amount of mitochondrial dehydrogenase; therefore, the assay is a reliable method to measure the metabolic activity of cells [41]. Results were analyzed using a Synergy HT Multi-Mode Microplate Reader (Bio-Tek Instruments, Winooski, VT, USA). The absorbance of the untreated cells was calculated as 100%, and the absorbance of all the other samples was expressed as percentage versus untreated cells.

#### 4.3.2. Sample Preparation for the Quantitative Evaluation of Ketogal and Ketorolac in CoEpi Cells

CoEpi cells were incubated in the presence of either 1, 2.5, or 5 mM ketorolac or ketogal. After different incubation times (24, 48, or 72 h), cells were washed with PBS, collected, and lysed in 0.3 mL of ice-cold RIPA buffer (50 mM Tris, 10 mM EDTA, 1% *v*/*v* TritonX100; pH 7.5). Lysates were clarified by centrifugation at 15,000× *g*, for 10 min, and the pellets were discarded. The supernatants were transferred into fresh tubes for reverse-phase HPLC analysis. Each experimental point was performed in triplicate.

#### 4.3.3. RP-HPLC Analysis

The reverse-phase HPLC procedure provided the separation and quantitation of ketogal and ketorolac. The amount of compounds in the cell extracts was expressed as nmol/mg protein. HPLC analysis was performed using an HP 1100 chromatograph system (Agilent Technologies, Palo Alto, CA, USA) equipped with a quaternary pump (model G1311A), a membrane degasser (G1379A), and a diode-array detector (DAD) (model G1315B) integrated into the HP1100 system. Data analyses were processed using a HP ChemStation system (Agilent Technologies). The analytical column was ZORBAX SB-Phenyl (250 × 4.6 mm, 5 µm; Agilent). The mobile phase consisted of 0.1% aqueous TFA and 0.1% TFA CH_3_CN 60/40 *w*/*w*, at a flow rate = 1.0 mL/min. The injection volume was 20 µL (Rheodyne, Cotati, CA, USA). The column effluent was monitored at 310 nm, referenced against an 800 nm wavelength. The quantitation of ketogal and ketorolac was performed using calibration curves obtained by analyzing standard solutions of compounds in the previously described chromatographic conditions. The linearity of the calibration curves was determined in a concentration range of 0.5–100 µM (r^2^ > 0.999).

#### 4.3.4. Extraction of Mitochondria

Mitochondria were isolated as previously reported [42]. The pellet, enriched of mitochondria, was finally resuspended in 0.25 mL resuspension buffer (250 mM sucrose, 15 mM K_2_HPO_4_, 2 mM MgCl_2_, 0.5 mM EDTA, 5% *w*/*v* BSA) and stored at −80 °C until use. Protein content was measured on a 50 μL aliquot.

#### 4.3.5. Mitochondrial Depolarization

Cells were incubated in PBS with 5 μg/mL JC-1 (Biotium Inc., Freemont, CA, USA), for 30 min at 37 °C, then washed and rinsed with 300 μL PBS. As reported [43], polarized mitochondria emitted a red fluorescence, while depolarized and damaged mitochondria emitted a green fluorescence. The depolarization and damage to mitochondria was associated with an increase in green/red fluorescence intensity. Red and green fluorescence units were measured using a Synergy HT Multi-Mode microplate reader, with 550 nm excitation/600 nm emission for red fluorescence, and 485 nm excitation/535 nm emission for green fluorescence. Fluorescence units were expressed as percentage of green-fluorescent (i.e., depolarized) mitochondria/red-fluorescent (i.e., polarized) mitochondria.

#### 4.3.6. Mitochondrial Reactive Oxygen Species (ROS)

Mitochondrial ROS were measured fluorometrically by incubating cells 10 min with 10 µM of MitoSOX Red (Invitrogen, Waltham, MA, USA), a superoxide-sensitive probe. Cell were washed and the red fluorescence, indicative of ROS generated within mitochondria, was read with a Synergy HT Multi-Mode microplate reader. Results were expressed as nmol/mg mitochondrial proteins.

#### 4.3.7. Thiobarbituric Acid Reactive Substances (TBARS)

TBARS formed as a byproduct of lipid peroxidation and measured as a marker of lipid damage, were detected spectrophotometrically by measuring adduct TBARS-TBA, which absorbs at a wavelength of 535 nm [44], in mitochondrial extracts with the TBARS Assay Kit (Cayman Chemicals, Ann Arbor, MI, USA), using a Synergy HT Multi-Mode microplate reader. Results were expressed as nmol/mg mitochondrial proteins.

#### 4.3.8. Intracellular Glutathione Measurement

Intracellular glutathione was measured as previously described [45]. The kinetics of the reaction was monitored for 5 min at 415 nm, using a Synergy HT Multi-Mode Microplate Reader. The subtraction of GSSG from total GSH gave the value of GSH. Results were expressed as nmol of glutathione/min/mg cellular protein, according to a titration curve prepared with serial dilutions (10 µM–0.1 nM) of 1:1 GSH + GSSG mix.

### 4.4. Animals

Male Swiss CD1 mice (30–35 g) of 10 weeks of age were purchased from Charles Rivers (Calco, Italy) and housed in cages in a room at 22 ± 1 °C on a 12/12 h light/dark cycle fed with a standard rodent chow diet and had ad libitum access to water. Each group of mice (*n* = 6) was treated for 5 days with either ketorolac (10 mg/kg/os), ketogal (16.3 mg/kg/os), or indomethacin (15 mg/kg/os), and a vehicle (carboxymethyl cellulose 0.5%). At the end of treatment, mice were fasted overnight for 12–14 h and then perfused for histological evaluations.

#### Ethics Statement

All experiments involving mice were carried out according to the ethical policies and procedures approved by the Institutional Committee on the Ethics of Animal Experiments (CSV) of the University of Naples “Federico II” and by the Ministry of Health (Approval no. 0084607, date 3 October 2014).

### 4.5. Histological Evaluations

Histological evaluations were performed on fasting mice deeply anesthetized with pentobarbitone (50 mg/kg, ip) and transcardially perfused with PBS, followed by 4% paraformaldehyde (PFA). Intestine and liver were collected and fixed in 4% buffered formaldehyde solution at 4 °C for 24 h. Samples were processed for wax embedding; serial sections (5 µm) were cut and stained with hematoxylin–eosin to highlight morphological alterations in the intestine and liver; periodic acid–Schiff (PAS) to underline mucin, glycogen, and glycoproteins; or Masson’s Trichrome staining to assess the presence of fibrosis, in accordance with Pagliuca et al. [46]. Sections were oxidized in 0.5% periodic acid solution for 10 min, rinsed in double-distilled water, and stained with Schiff’s reagent in the dark for 45 min. The reaction was blocked by repeated washing in 2.5% sodium bisulfite in 0.05 N HCl [47]. To perform Masson’s trichrome, liver sections were stained in iron hematoxylin solution for 10 min and placed in Biebrich’s scarlet fuchsin solution for 15 min, differentiated in phosphomolybdic acid solution, transferred directly to an aniline blue solution for 10 min, and further differentiated in 1% glacial acetic acid solution for 1 min. Observations of intestinal lesions were graded according to Park’s Scale [48]. Intestinal villus length measurements were made on randomly selected digital image photographs of untreated and treated mice and analyzed using the software Image J 1.45.

### 4.6. Statistical Analyses

Data were analyzed using one-way ANOVA followed by post hoc Bonferroni’s multiple comparison test to determine the significance of differences between groups. Results are reported as mean ± SEM of at least two-to-three independent experiments, performed in triplicate. Statistical significance was accepted at *p* < 0.05.

## 5. Conclusions

In conclusion, we can undoubtedly confirm that ketogal has a better safety and reliability profile than ketorolac. Interestingly, in the colon, ketogal was significantly less toxic than its parent drug. Notably, the prodrug induced only a 10% reduction in CoEpi cell viability, and this decrease was not due to any mitochondrial damage or alteration. Remarkably, after in vitro treatment with ketogal, mitochondrial oxidative stress parameters were comparable to control samples. Furthermore, in ketogal-treated mice, a significant improvement in the morphology of both the upper GI tract and the liver was observed, which appeared to be similar to that of untreated animals. With this information, ketogal could replace ketorolac and, at the same time, reduce the use of drugs, such as PPIs, that are normally prescribed to mitigate the side effects of NSAIDs. The use of a single drug could be the best alternative in terms of health and public spending.

## Data Availability

The data presented in this study are available in article.
